# Whole-blood transcriptomic signatures induced during immunization by chloroquine prophylaxis and *Plasmodium falciparum* sporozoites

**DOI:** 10.1038/s41598-019-44924-7

**Published:** 2019-06-10

**Authors:** Tuan M. Tran, Else M. Bijker, Mariëlle C. Haks, Tom H. M. Ottenhoff, Leo Visser, Remko Schats, Pratap Venepally, Hernan Lorenzi, Peter D. Crompton, Robert W. Sauerwein

**Affiliations:** 1 Division of Infectious Diseases, Department of Medicine, Indiana University School of Medicine, Indianapolis, IN USA; 2 Department of Medical Microbiology and Radboud Center for Infectious Diseases, Radboud University Medical Center, Nijmegen, The Netherlands; 3 Department of Infectious Diseases, Leiden University Medical Center, Leiden, The Netherlands; 4 Department of Infectious Diseases, J. Craig Venter Institute, Rockville, Maryland USA; 5 Laboratory of Immunogenetics, National Institute of Allergy and Infectious Diseases, National Institutes of Health, Rockville, MD USA

**Keywords:** Predictive markers, Malaria

## Abstract

A highly effective vaccine that confers sterile protection to malaria is urgently needed. Immunization under chemoprophylaxis with sporozoites (CPS) consistently confers high levels of protection in the Controlled Human Malaria infection (CHMI) model. To provide a broad, unbiased assessment of the composition and kinetics of direct *ex vivo* human immune responses to CPS, we profiled whole-blood transcriptomes by RNA-seq before and during CPS immunization and following CHMI challenge. Differential expression of genes enriched in modules related to T cells, NK cells, protein synthesis, and mitochondrial processes were detected in fully protected individuals four weeks after the first immunization. Non-protected individuals demonstrated transcriptomic changes after the third immunization and the day of treatment, with upregulation of interferon and innate inflammatory genes and downregulation of B-cell signatures. Protected individuals demonstrated more significant interactions between blood transcription modules compared to non-protected individuals several weeks after the second and third immunizations. These data provide insight into the molecular and cellular basis of CPS-induced immune protection from *P*. *falciparum* infection.

## Introduction

Malaria afflicts over 200 million people globally, causing 435,000 deaths in 2017, primarily in young children suffering from *Plasmodium falciparum* malaria in sub-Saharan Africa^1^. Although these numbers reflect substantial reductions in morbidity and mortality over the past two decades due to successful malaria control efforts, recent progress has plateaued or reversed in some regions^1^. Thus, further progress towards malaria eradication will require the development of new tools such as a highly effective malaria vaccine that ideally would target the parasite during the asymptomatic, pre-erythrocytic stage of its life cycle^2,3^.

Sterile protection against *P*. *falciparum* infection has been consistently and efficiently achieved in malaria-naïve adults via immunization with infectious sporozoites under the cover of antimalarial chemoprophylaxis^4,5^. This approach, termed chemoprophylaxis with sporozoites (CPS), provides a unique opportunity to elucidate the immune mechanisms underlying protective responses against pre-erythrocytic stages. Prior studies focusing on immune responses in peripheral blood mononuclear cells (PBMCs) have implicated Th1 and cytotoxic T-cell responses, including γδ T cells and *P*. *falciparum*-specific CD8+ and CD4+ T cells, in CPS-induced immunity^6,7^. However, genome-wide host responses to CPS have not been investigated.

To provide a broad, unbiased assessment of the composition and kinetics of direct *ex vivo* immune responses to CPS, we performed whole-blood transcriptome profiling in malaria-naïve adults before and during CPS and after challenge with controlled human *P*. *falciparum* malaria infections (CHMI). Our aims were to define the most specific time points for whole-blood transcriptome profiling during CPS and to explore CPS-induced molecular signatures associated with protection.

## Results

Transcriptome profiling was performed by RNA-seq using whole-blood samples collected at 15 time points before and after each CPS immunization dose and CHMI challenge as well as immediately before treatment with anti-malarials (Fig. S1). The protected (P) and not protected (NP) groups each had five subjects, with four (80%) women in the P group and one woman (20%) in the NP group. Principal components analysis (PCA) revealed variation along PC1 that was best explained by a batch and/or subject effect (Fig. S2a–c). Variation along PC2 was likely explained by immunization responses in two protected subjects (Fig. S2a–c). To minimize uninformative variation, sex-specific genes were removed from the analysis, normalization factors were calculated to scale the raw library sizes, and differential gene expression analysis included subject (which accounts for almost all batch effects) as a co-variate as per Methods.

**Figure 1:**
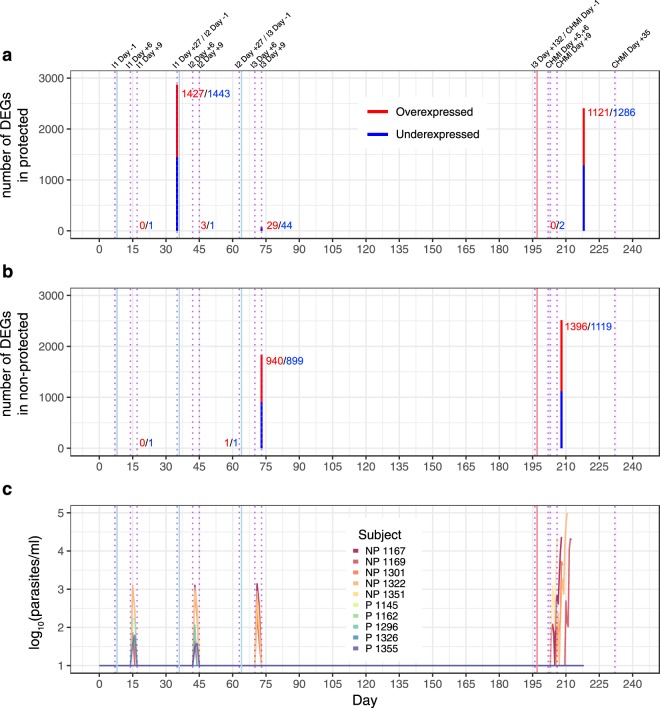
Number of differential expressed genes (DEGs) having a false discovery rate threshold <25% (no fold-change cut-off) at immunization (I) or challenge (CHMI) time points for **(a)** protected (P) subjects relative to their baseline, **(b)** non-protected (NP) subjects relative their baseline, and **(c)** timing with regard to detectable parasitemia. Blue lines and dotted purple lines indicate vaccination and RNA collection time points, respectively. Day of malaria treatment post-CHMI occurred on different days in the NP group but was treated as a single time point for analysis and for graphical representation.

For two out of five subjects in each group, baseline gene expression values were unavailable and were imputed in a gender-specific manner (Fig. S1; refer to Methods). Differentially expressed genes (DEGs; false discovery rate [FDR] threshold of <25%) were determined at each time point relative to baseline to assess the transcriptomic changes induced by immunization within P and NP groups (Fig. 1). Within the P group, the greatest number of DEGs occurred at I1 Day +27 (I2 Day −1; 2870 DEGs) and just prior to anti-malarial treatment (study day +218; 2407 DEGs; Fig. 1a). Within the NP group, the greatest number of DEGs occurred at I3 Day +9 (1839 DEGs) and just prior to anti-malarial treatment (variable days post-CHMI; 2515 DEGs; Fig. 1b). The majority of NP DEGs were upregulated relative to baseline (Fig. 1b). To assess the transcriptomic changes associated with protection by CPS immunization, we directly compared the P versus NP groups but did not observe any DEGs at baseline or any post-immunization time point using a FDR threshold of <25%. Of note, detectable parasitemia occurred in all NP subjects and in four P subjects between Day +6 and Day +9 post-immunization (Fig. 1c), which likely influenced the blood transcriptome at these time points. Results of the differential gene expression analysis for each comparison at each time point can be found in the Supplementary Materials (Supplementary Dataset 1).

**Figure 2:**
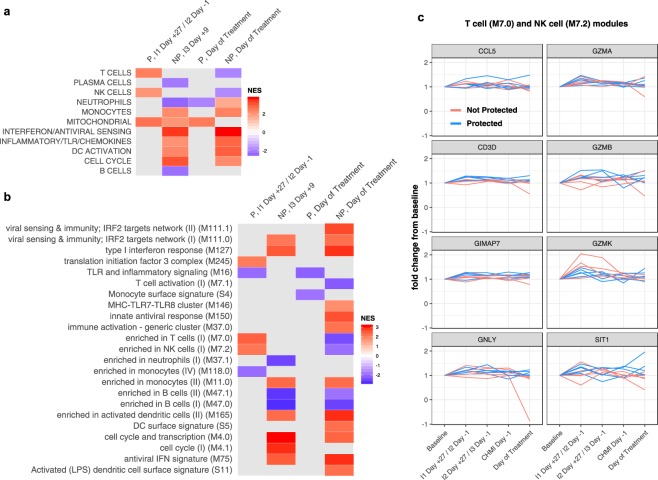
Gene set enrichment analysis at timepoints with significant differential gene expression in Fig. 1 using (**a**) high-level and (**b**) low-level blood transcription modules (BTMs). Normalized enrichment scores (NES) are determined relative to pre-vaccination baseline. Only BTMs with Benjamini-Hochberg adjusted P values < 0.05 are shown. (**c**) Fold-change expression for the top leading-edge genes among the two BTMs with the highest NES for the protected (P) group at I1 Day +27/I2 Day −1. Values for the not protected (NP) group are shown for comparison. DC = dendritic cell; IFN = interferon; IRF2 = interferon-regulatory factor 2; LPS = lipopolysaccharide; MHC = major histocompatibility complex; TLR = toll-like receptor.

To provide biological and functional context for the immunization-induced transcriptional changes observed at specific time points in Fig. 1a,b, we performed gene set enrichment analysis (GSEA)^8^ using DEGs (FDR < 25%) ranked by fold-change as input and blood transcription modules^9,10^ (BTMs) as gene sets. Within the P group, significant upregulation of genes within T cells, natural killer (NK) cells, and mitochondrial modules was observed at I1 Day +27/I2 Day −1 relative to baseline using high-level annotation modules (Fig. 2a) that corresponded to upregulation of T cells (M7.0), NK cells (M7.3), and translation initiation (M245) modules and downregulation of TLR and inflammatory signaling (M16) and monocyte (M118.0) modules in low-level annotations (Fig. 2b). In GSEA, leading edges are genes in the gene set that appear in the ranked list at or before the point at which the running sum reaches it maximum deviation from zero^8^. The top leading edges among the BTMs with the highest normalized enrichment scores (M7.0, M7.3) for the protected group at I1 Day +27/I2 Day −1 were consistently upregulated in protected individuals at I1 Day +27/I2 Day −1, as expected, but were not sustained above baseline at subsequent time points (Fig. 2c, blue lines). On the day of treatment, upregulation of mitochondrial genes and downregulation of neutrophils genes were observed in the P group (Fig. 2a). By contrast, within the NP group, there was broad upregulation of innate cell lineages, interferon, pro-inflammatory, and cell cycle signatures and downregulation of B-cell signatures at I3 Day +9 and on the day of treatment when compared to baseline (Fig. 2a,b), results that corresponded to patent parasitemia occurring just prior to and at these two respective timepoints (Fig. 1c).

**Figure 3:**
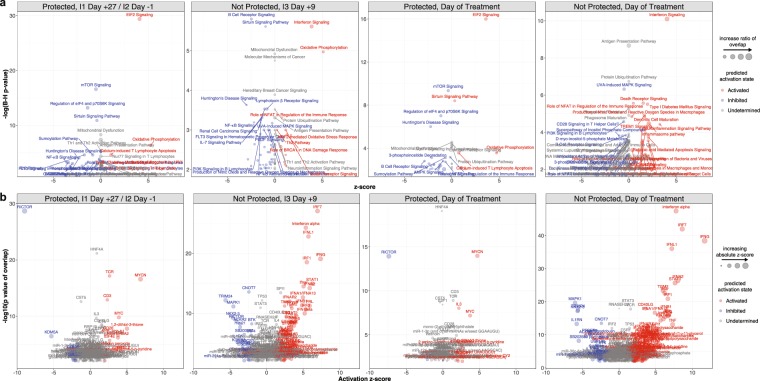
(**a**) Ingenuity pathways analysis and (**b**) upstream regulator analysis at timepoints with significant differential gene expression in Fig. 1. Z-scores that were unable to be determined were set to 0.

Ingenuity pathways analysis (IPA) applied to DEGs (FDR < 25%) at transcriptionally active time points in Fig. 1a,b predicted significant activation of canonical pathways related to protein synthesis (EIF2 signaling) and mitochondrial activity (oxidative phosphorylation) and upstream activation of T cell regulators (CD3 and T-cell receptor [TCR]) within the P group at I1 Day +27/I2 Day −1 and on the day of treatment relative to baseline (Fig. 3a,b), results which were consistent with GSEA. However, IPA also predicted activation of the upstream regulators of the MYC-family proto-oncogene transcription factors (MYCN and MYC) and inhibition of RICTOR, which is part of a protein complex that regulates cell growth by integrating nutrient-derived signals (Fig. 3b). The molecular signatures of CPS immunogenicity observed in the P group at I1 Day +27/I2 Day −1 may be partially explained by modestly significant changes in lymphocyte composition, as the proportions of CD4+ T cells increased above baseline at the corresponding time point after the first immunization (Fig. S3). Within the NP group, IPA predicted activation of interferon signaling and oxidative phosphorylation pathways and inhibition of B-cell receptor and sirtuin signaling pathways at I3 Day +9, whereas several interferon- and innate-related pathways were predicted to be activated on the day of treatment (Fig. 3a). Upstream regulator analysis predicted significant activation of several interferon-related genes in the NP group at both I3 Day +9 and on the day of treatment (Fig. 3b). Taken together, the enrichment and pathways analyses suggest activation and differentiation of T-cells after initial immunization and challenge in P subjects and a robust interferon response to parasitemia in NP subjects.

**Figure 4:**
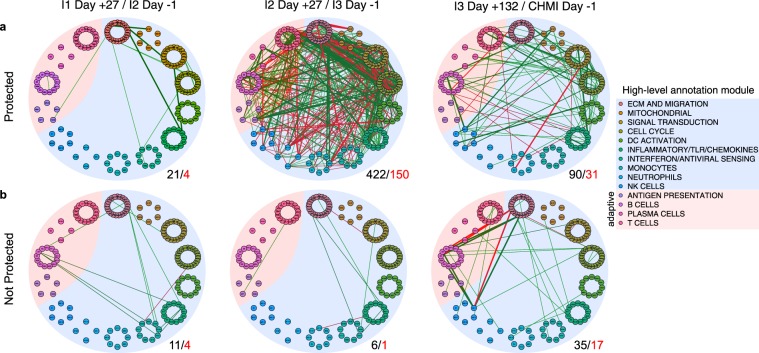
Blood transcription module correlation networks in (**a**) protected and (**b**) non-protected subjects. Data derived from RNA-seq performed one day prior to second vaccination (I1 Day +27/I2 Day −1), one day prior to third vaccination (I2 Day +27/I3 Day −1), and one day prior to challenge (I3 Day +132/CHMI Day −1). Small circles represent low-level annotation modules, which are grouped by color into larger circles representing high-level annotation modules. Width of edge is proportional to Pearson correlation coefficient between two connected modules. Green edges indicate positive correlations, and red edges indicate negative correlations. Only correlations with an absolute Pearson coefficient >0.8 and false discovery rate <25% are shown. Not all edges between members of the same high-level annotation module could be shown. The number of significant edges for all modules (black) and adaptive modules (red) are shown to the lower-right of each network.

To evaluate possible biological interactions between immune components post-immunization, we correlated BTM expression at I1 Day +27/I2 Day −1; I2 Day +27/I3 Day −1; and I3 Day +132/CHMI Day −1. These timepoints were chosen to assess post-immunization dose responses not confounded by potential parasitemia. We observed more interactions between BTMs in P subjects than NP subjects at all three time points, with a 20-fold increase in significant correlations post-immunization dose #2 relative to post-immunization dose #1 in P subjects (Fig. 4). Notably, a greater number of interactions involving adaptive modules were observed in P subjects relative NP subjects at I3 Day −1 and CHMI Day −1 (Fig. 4).

**Figure 5:**
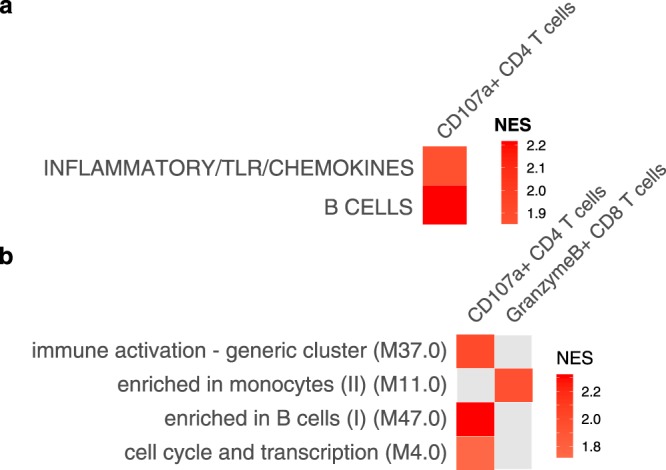
Gene set enrichment analysis of correlations between gene expression and later adaptive immune responses using **(a)** high-level and **(b)** low-level blood transcription modules. Expression values for each of 20,525 genes at I1 Day +27/I2 Day −1 (see Fig. S1) were correlated with the percentage of CD107a + CD4+ T cells or CD8+ T cells producing granzyme B after re-stimulation with *P*. *falciparum*-infected erythrocytes. Re-stimulated cells were PBMCs obtained a day before challenge (CHMI Day −1). Enrichment analysis were performed on correlations with P < 0.05 and ranked by Spearman rho. Only modules with Benjamini-Hochberg adjusted P values < 0.05 are shown. NES = normalized enrichment score.

In the parent CPS trial that included the current study, cytotoxic T cell responses to *P*. *falciparum* were associated with CPS-induced protection^7^. Recent studies have shown that whole-sporozoite vaccination of humans can elicit antibodies specific for *P*. *falciparum* circumsporozoite surface protein (CSP) that can prevent malaria infection in mouse models^11,12^. To assess early, CPS-induced molecular correlates of these potentially protective adaptive immune responses, we correlated expression values for each of the 20,525 most variable genes during the only two early, post-immunization time points that were not confounded by parasitemia (I1 Day +27 and I2 Day +27) with previously published data on the proportion of *in vitro* re-stimulated, *P*. *falciparum*-specific CD107a + CD4 T cells or granzyme B + CD8 T cells^7^ and IgG antibodies to CSP^13^ determined at 132 days after the third immunization (CHMI Day −1) in all subjects in the current study (n = 10). Genes measured at I1 Day +27 that significantly correlated (unadjusted P value < 0.05) with CD107a + CD4 T cells on CHMI Day −1 were enriched for genes within the inflammatory/TLR/chemokines and B cells high-level modules (Fig. 5a) and the M4.0, M37, and M47.0 low-level modules (Fig. 5b). Among the genes within the module with the strongest correlation, M47.0 (enriched in B cells [I]), expression of *E2F5*, *TSPAN13*, *PCDH9*, *PTPRK*, *FCRL2*, and *CD24* were the strongest positive correlates (r > 0.70, adjusted P value < 0.20) of subsequent CD107a + CD4 T cell responses (Fig. S4). Genes measured at I1 Day +27 that significantly correlated with granzyme B + CD8 T cells at CHMI Day −1 were enriched for genes within the M11.0 (enriched in monocytes [II]) low-level module only (Fig. 5b). Genes measured at I2 Day +27 that correlated with CSP antibodies at CHMI Day −1 showed downregulated enrichment of M37.0 (immune activation – generic cluster; normalized enrichment score = −1.60; adjusted P value = 0.04). No significant enrichment of BTMs was detected for I1 Day +27 genes that correlated with CSP antibodies and for I2 Day +27 genes that correlated with either of the T-cell responses.

## Discussion

Systems vaccinology approaches employing blood transcriptomics have been applied to vaccines against bacteria^14^, viruses^10^, and, more recently, malaria^9,15^. In this study, we aimed to determine the most informative time points to detect differences in the blood transcriptome during and after CPS immunization in protected and non-protected vaccinees and to correlate these molecular signatures to adaptive immune responses.

Although we determined differential expression at the individual gene level to provide a quantitative measure of transcriptional activity, we refrain from concluding that the differential expression of any specific gene is associated with protection or susceptibility after CPS given the lack of validation and the possibility of a Type I error. However, we have more confidence in providing an assessment of transcriptionally active time points based on module enrichment and pathways analysis, as this relies on relative representation of biologically meaningful gene sets rather than single genes.

In the current study, we detected robust changes in the blood transcriptome four weeks after the first immunization that were unique to protected subjects. These changes were detected just prior to the second immunization during a period in which the subjects were aparasitemic, and presumably reflect a “steady state” in terms of peripheral blood cell composition. T-cell, NK-cell, protein synthesis, and mitochondrial signatures were detected by enrichment and pathways analysis, suggesting induction of cytotoxic cellular responses after a single CPS immunization. Although no signatures were detected in protected subjects four weeks after the second immunization (I2 Day +27), or 132 days after the third immunization (CHMI Day −1), we did detect similar signatures three weeks after challenge by CHMI (day of treatment), suggesting an ongoing T cell memory response after sporozoite exposure and apparent clearance of antigen. Why such signatures can be detected weeks after perturbation will need further characterization at the cellular level to determine malaria specificity.

In non-protected subjects, transcriptomic changes were detected after the third immunization and on the day of treatment, with both timepoints corresponding to recently cleared or patent parasitemia in all five subjects. Not surprisingly, upregulation of interferon and innate inflammatory signatures and downregulation of B-cell signatures at these time points were consistent with previous transcriptomic analyses of CHMI^16,17^. Recent analyses of whole-blood transcriptomes in malaria-exposed Tanzanian volunteers^18^ and RTS,S vaccinees^9^ who underwent CHMI revealed marked transcriptional changes within one week of inoculation. In contrast, we did not detect any differential gene expression within this time frame, which may be due to differences in prior malaria exposure in the case of the Tanzanian study or relatively smaller antigen load versus lack of statistical power in the current study.

Similarly, unlike recent systems analyses of the response to the RTS,S vaccine^9,15^, we did not detect differential gene expression relative to baseline within one week after each immunization dose. This difference may reflect a smaller sample size in the current study or a more robust immune perturbation elicited by a recombinant antigen vaccine (RTS,S) containing the strong adjuvant AS01E relative to immunization by infectious mosquito bites. Indeed, the transcriptomic differences also appear to be qualitative, with RTS,S vaccination inducing robust innate and B-cell responses^9,15^ and CPS immunization inducing cytotoxic cellular responses, and may reflect the different mechanisms by which each vaccination regimen confers protection.

Evidence for enhanced complexity of immune interactions weeks after immunization in protected individuals implies that cross talk between adaptive and innate cells may be required to achieve sterile immunity. These findings require validation in a larger CPS study and evaluation in other effective whole-sporozoite vaccination approaches to determine generalizability. More functional studies may also address the antigen-specificity of these interactions and how robust activity in peripheral immune cells can confer sterile immunity.

The current study assessed 15 time points during and after CPS immunization to determine the time points that might provide the highest signal for blood transcriptome profiling during CPS. However, the study was limited by the small number of individuals per group, which reduced our ability to detect any CPS-induced molecular signatures of protection. In addition, we only examined post-CHMI responses to homologous challenge, which may further limit generalizability to studies that evaluate CPS-induced protection to heterologous challenge^19^. Nevertheless, transcriptomic analysis of whole blood after CPS vaccination can provide important biological insights into the protective immune responses against *P*. *falciparum* infection. The current study can serve as a guidepost to better define the time course of transcriptomic changes detectable in peripheral blood in recipients of whole sporozoite vaccination approaches.

## Methods

### Human ethics statement

Subjects provided written, informed consent prior to screening and enrollment. The Central Committee for Research Involving Human Subjects of the Netherlands approved the study (NL33904.091.10), which complied with the Declaration of Helsinki and good clinical practice, including data monitoring.

### Clinical trial design and procedures

Details of the single-center, double-blind study conducted at the Leiden University Medical Center from April 2011 to April 2012 have been described previously^7^. Briefly, healthy subjects aged 18–35 years with no prior history of malaria were randomized into four groups. Subjects received three CPS immunizations at 28-day intervals with varying doses of NF54 *P*. *falciparum*–infected versus uninfected mosquitoes as follows: five subjects were each exposed to bites from 15 infected mosquitoes three times (Group 1), 10 subjects were each exposed to bites from 10 infected and five uninfected mosquitoes three times (Group 2), 10 subjects were each exposed to bites from five infected and 10 uninfected mosquitoes three times (Group 3), and five control subjects were exposed to bites from 15 uninfected mosquitoes three times (Group 4). Nineteen weeks after the final immunization (15 weeks after the last chloroquine dose), all subjects were challenged by exposure to bites from five mosquitoes infected with the homologous NF54 *P*. *falciparum* strain, according to published protocols^4^. Protection was defined as absence of parasitemia by daily monitoring by peripheral blood thick smears for 21 days after challenge^7^. However, in practice, in this trial, all protected subjects were also PCR negative for *P*. *falciparum* until Day 21. The current study focuses on comparisons between protected and non-protected subjects who underwent CPS immunization in Group 3, which was chosen due to the balanced distribution of five protected and five non-protected subjects.

### Blood collection

Peripheral whole blood was collected in PAXgene blood RNA tubes (PreAnalytiX) at the following time points (Fig. S1): before initiation of chloroquine prophylaxis (baseline); +6, +9, and +27 days from immunization 1 (I1); +6, +9, and +27 days from immunization 2 (I2); +6, +9, and +132 days from immunization 3 (I3); and +5, +6, +9, and +35 days from challenge by CHMI. Whole-blood RNA was also collected on the day of treatment (immediately before receipt of anti-malarials), which occurred 10 to 15 days after CHMI for NP subjects and 21 days after CHMI for all P subjects (Fig. S1). Whole-blood RNA could not be collected at baseline for three subjects and at I2 Day +27 for one subject. Peripheral blood mononuclear cells (PBMCs) and plasma were also collected before initiation of chloroquine prophylaxis (baseline) and at I1 Day +27/I2 Day −1; I2 Day +27/I3 Day −1; and I3 Day +27; one day prior to CHMI; and 35 days after CHMI.

### Generation of RNA-seq data

Total RNA was extracted from whole blood using the PAXgene Blood miRNA Kit (Qiagen) and then treated with ScriptSeq™ Complete Gold Kit (Blood) (Epicentre) for removal of adult globin mRNAs as well as cytoplasmic and mitochondrial rRNA transcripts. Remaining RNA was used as template to prepare RNA sequencing libraries with PrepX RNA-Seq library preparation kit (Wafergen). Libraries were grouped and sequenced multiplexed to generate an average of 100 million reads per library as follows: 45 libraries were sequenced in an Illumina HiSeq 2000 machine to generate 100 bp paired-end reads, 15 libraries were sequenced with Illumina NextSeq 500 to generate 150 bp paired-end reads and the remaining 159 libraries were sequenced with Illumina NextSeq 500 to generate 75 bp paired-end reads. Illumina paired-end sequences from all 219 samples were cut to retain only the 5′-most 75 bases and then processed with Trimmomatic^20^ to trim any primer/adapter sequence and low-quality bases using a Phred quality score cutoff of 12. Only sequences longer than 25 bases were mapped simultaneously to the human genome (GRCh38, version 25, GENCODE) and the *P*. *falciparum* genome (Pf3D7, version 29 from PlasmoDB) using HISAT2^21^. Transcript abundance was determined by stringtie^21^. Libraries could not be generated for one baseline sample (subject 1169). Samples from the same subject underwent the same library preparation steps. As much as possible, we ensured that all samples for the same subject were included in the same library preparation batch. Sequencing data is available on the dbGAP under the accession number phs001346.v1.p1.

### Gene expression analysis

Gene expression analysis of RNA-seq read counts was performed using edgeR (version 3.22.4)^22^. For the four subjects with missing baseline samples (two protected and two non-protected), baseline expression values for each gene were imputed using the median count of available baseline samples in a gender-specific manner. Samples were reduced to include only those from baseline and the timepoint of interest prior to gene pre-filtering, normalization, and differential gene expression (DGE) analysis. Male-specific genes on the Y chromosome, X-inactivated genes, and low-expression genes (genes with less than 5 counts per million (CPM) in at least 5 samples) were removed prior to TMM normalization. For each clinical outcome (protected [P] or not protected [NP]), we compared the effects of vaccination within subjects while taking into account subject by using the following model formula: ~0 + Timepoint + Subject, in which Timepoint was the main effect. Differences in sex, age, and, in most instances, batch were accounted for by the subject effect. Day of malaria treatment post-challenge occurred on different days in the NP group and on day 218 of the study in the P group (Fig. S1) but was treated as one time point for analysis. DGE analysis was performed by fitting counts for each gene to a quasi-likelihood negative binomial generalized log-linear model using the glmQLFit function in edgeR for the following *within* outcome contrasts, where Timepoint represents the vaccination time point and baseline represents the pre-CPS vaccination baseline:

$$\begin{array}{c}{{\rm{P}}}_{{\rm{Timepoint}}}-{{\rm{P}}}_{{\rm{Baseline}}}\\ {{\rm{NP}}}_{{\rm{Timepoint}}}-{{\rm{NP}}}_{{\rm{Baseline}}}\end{array}$$

Comparisons between outcomes were also performed using the model formula ~0 + Group + Subject, in which Group was a combined parameterization of clinical outcome with timepoint and the following contrasts:

$$({{\rm{P}}}_{{\rm{Timepoint}}}-{{\rm{P}}}_{{\rm{Baseline}}})-({{\rm{NP}}}_{{\rm{Timepoint}}}-{{\rm{NP}}}_{{\rm{Baseline}}})$$

Lastly, between group comparison of non-imputed baseline samples was performed using the model formula ~0 + Outcome + Batch, in which Outcome represented clinical outcome and Batch represented the library sequencing batch, and the P_baseline_ − NP_baseline_ contrast. Geneset enrichment analysis^8^ was performed using pre-ranked gene lists (DEGs with FDR < 25%, sorted by decreasing log fold change) derived from edgeR results for each contrast with the fgsea function from the fgsea package^23^ (version 1.60) and blood transcription modules^9,10^ (BTMs) as gene sets. Ingenuity canonical pathways and upstream regulator analyses were performed using DEGs with FDR < 25%.

### Module correlation network analysis and molecular correlates of immunogenicity

Summarized expression values for BTMs were calculated as the mean of z-score-transformed expression values for all genes within a module. Pearson correlations between summarized BTM expression within pre-vaccination or pre-CHMI timepoints were determined and plotted using the qgraph package^24^ (version 1.5). Gene expression values from select pre-vaccination time points were also correlated with previously published data on cytotoxic T cell responses at CHMI Day −1 from the same trial^7^. Gene set enrichment analysis was then performed on genes ranked by Spearman correlation coefficients using BTMs with a minimum size of 10 genes.

### Immunological methods

Immunological methods have been previously described^6,7,13^. Briefly, peripheral blood mononuclear cells collected at specific time points (see “Blood collection” above) were re-stimulated with either uninfected erythrocytes or *P*. *falciparum*-infected erythrocytes in culture for 24 h. Stimulated cells were assessed for expression of the degranulation marker CD107a, the cytotoxic molecule granzyme B, and the cytokine interferon γ (IFN-γ) by CD4+ T cells, CD8+ T cells, γδ T cells, CD56+ natural killer cells by flow cytometry^6,7^. A viability stain was included in all flow cytometry panels, and only single, living PBMCs were used in the analysis. Values from uninfected erythrocyte-cultured PBMCs were used for subtraction of background from infected erythrocyte-stimulated values. PBMCs cultured with uninfected erythrocytes were also used for immunophenotyping to determine the relative proportions of lymphocyte subsets at each time point. IgG antibodies specific to *P*. *falciparum* CSP were determined in plasma by a standardized enzyme-linked immunosorbent assay as previously described^13^.

## Supplementary information

Supplementary Materials

Supplementary Dataset 1

## Data Availability

Sequencing data will be available on the dbGAP under the accession number phs001346.v1.p1.
