# Supplementary material for: Whole-blood transcriptomic signatures induced during immunization by chloroquine prophylaxis and Plasmodium falciparum sporozoites
Source: Sci Rep. 2019 Jun 10;9:8386. doi: 10.1038/s41598-019-44924-7 (PMC6557840; doi:10.1038/s41598-019-44924-7)
Supplement: Supplementary file 1 — Supplementary Materials [file 41598_2019_44924_MOESM1_ESM.docx]

**Supplementary Materials for:**

**Whole-blood transcriptomic signatures induced during immunization by chloroquine prophylaxis and *Plasmodium falciparum* sporozoites**

Tuan M. Tran^1^, Else Bijker^2^, Mariëlle C. Haks^3^, Tom H.M. Ottenhoff^3^, Leo Visser^3^, Remko Schats^3^, Pratap Venepally^4^, Hernan Lorenzi^4^, Peter D. Crompton^5*^, Robert Sauerwein^2*^

^1^Division of Infectious Diseases, Department of Medicine, Indiana University School of Medicine, Indianapolis, IN, USA

^2^Department of Medical Microbiology, Radboud University Medical Center, Nijmegen, The Netherlands

^3^Department of Infectious Diseases, Leiden University Medical Center, Leiden, The Netherlands

^4^Department of Infectious Diseases, J. Craig Venter Institute, Rockville, Maryland, USA

^5^Laboratory of Immunogenetics, National Institute of Allergy and Infectious Diseases, National Institutes of Health, Rockville, MD, USA

*Correspondence to [pcrompton@niaid.nih.gov](mailto:pcrompton@niaid.nih.gov) or [robert.sauerwein@radboudumc.nl](mailto:robert.sauerwein@radboudumc.nl)

**
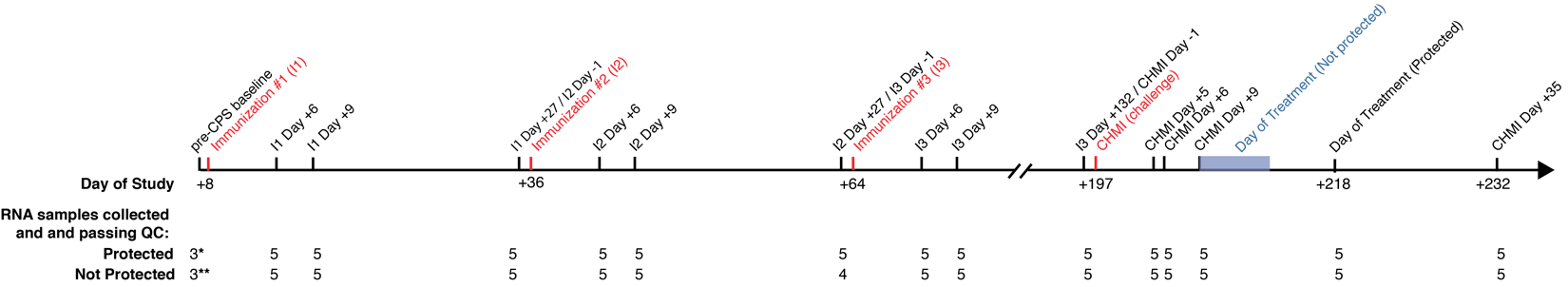
**

**Figure S1.** Study Design. Overview of chemoprophylaxis with sporozoite (CPS) immunization regimen with controlled-human malaria infection (CHMI) challenge. *Two RNA samples were not available. **One sample was not available, and one sample yield insufficient libraries for analysis. For subjects with missing baseline samples, baseline expression values for individual genes were imputed using the median count of available baseline samples in a gender-specific manner for downstream analyses.


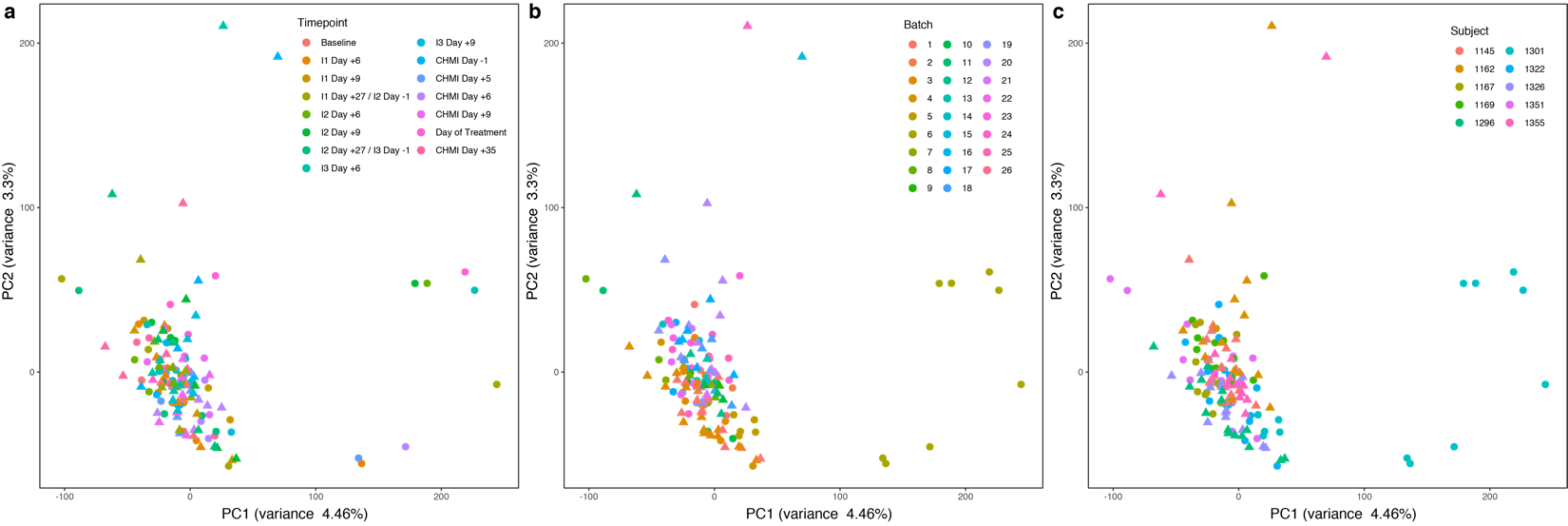


**Figure S2.** Principal components analyses with samples colored by (**a**) timepoint, (**b**) library preparation batch, and (**c**) study subject. Spheres and triangles represent samples from subjects not protected and protected from malaria, respectively.

**Supplementary Dataset 1.** Differentially expressed genes for each comparison at each time point using a false discovery rate threshold of <25%. Comparisons were made relative to pre-immunization baseline *within* protected or not protected groups.

<https://iu.box.com/v/CPStranscriptomicsdegs>

[note to copy editor: the above link should be replaced with the journal’s link to Dataset 1 if published.]

**
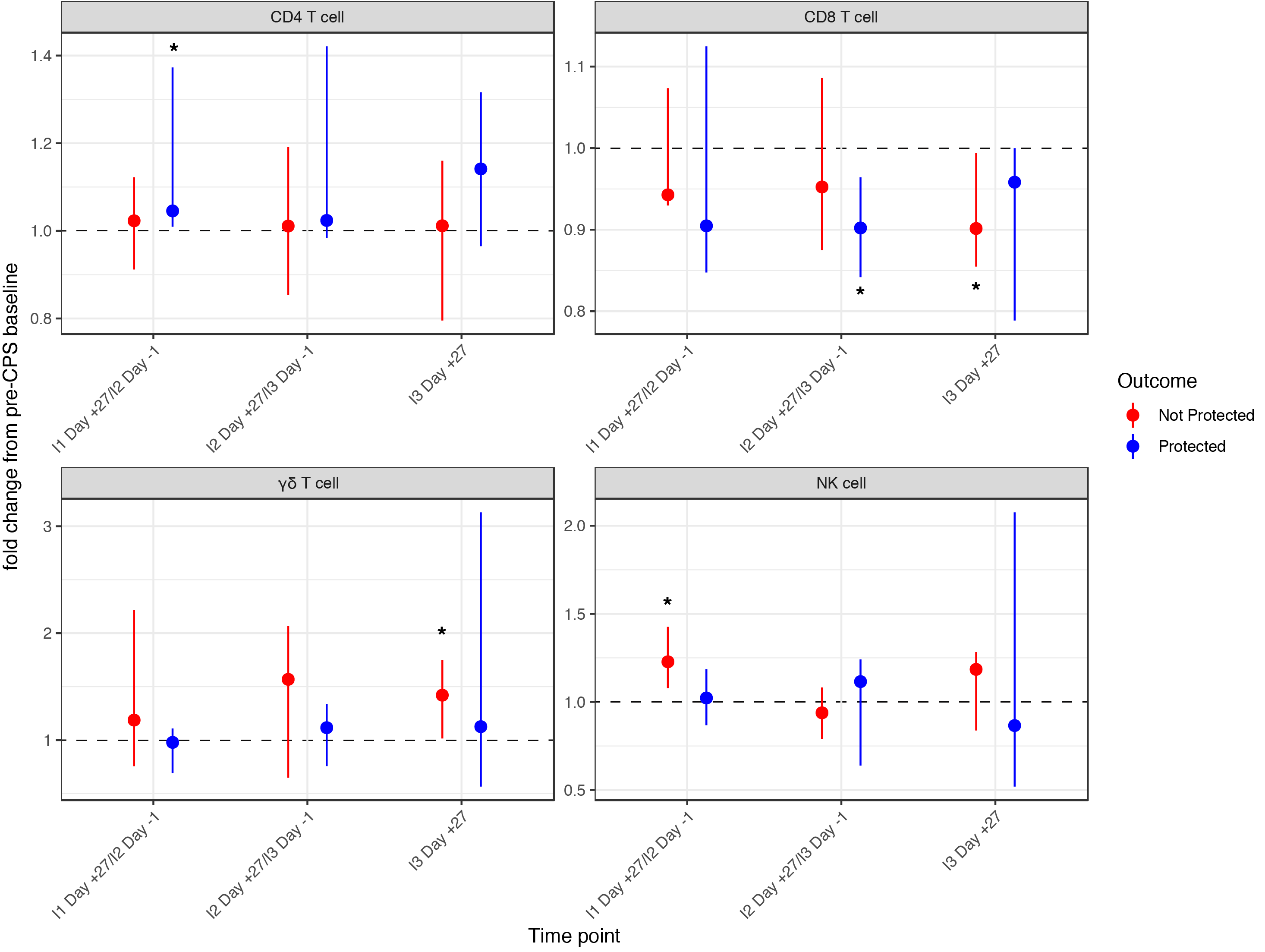
**

**Figure S3.** Fold-change of cell proportions over pre-immunization baseline for T-cell subsets and NK cells.

Peripheral blood mononuclear cells collected 8 days prior to the first CPS immunization (baseline) and at the indicated timepoints were cultured with uninfected erythrocytes for 24 h and immunophenotyped to determine the proportion of CD4+, CD8+, γδ T-cell receptor +, and CD56+ NK cells among viable CD3+ and CD56+ lymphocytes. Fold-change was calculated by dividing each time point by the baseline value. Points represent group medians at each time point and vertical lines extend from the 2.5 and 97.5 quantiles. Time points that significantly differed from baseline for any of the cell subsets for either outcome by Wilcoxon test are shown by an asterisk (Holm-adjusted P value < 0.05).

**
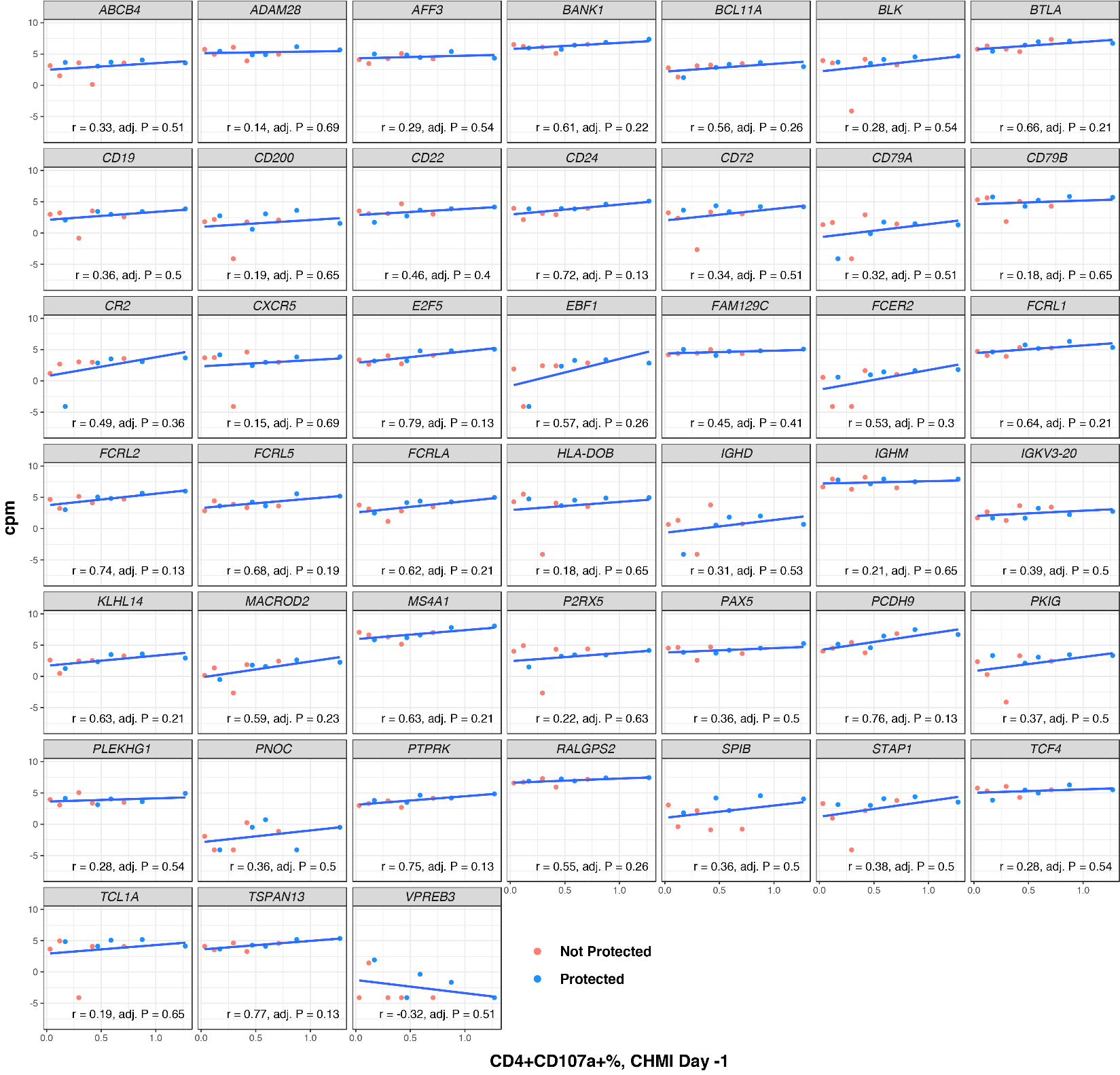
Figure S4.** Correlations between expression of individual genes within the M47.0 module “enriched in B cells (I)” at I1 Day +27 / I2 Day -1 and percentage of CD107a+ CD4+ T cells one day prior to challenge (CHMI). Pearson coefficient (r) and Benjamini-Hochberg-adjusted P values are shown.
